# *Corynebacterium diphtheriae *invasion-associated protein (DIP1281) is involved in cell surface organization, adhesion and internalization in epithelial cells

**DOI:** 10.1186/1471-2180-10-2

**Published:** 2010-01-05

**Authors:** Lisa Ott, Martina Höller, Roman G Gerlach, Michael Hensel, Johannes Rheinlaender, Tilman E Schäffer, Andreas Burkovski

**Affiliations:** 1 Friedrich-Alexander-Universität Erlangen-Nürnberg, Lehrstuhl für Mikrobiologie, Staudtstr. 5, 91058 Erlangen, Germany; 2 Mikrobiologisches Institut des Universitätsklinikums Erlangen, Wasserturmstr. 3-5, 91054, Erlangen, Germany; 3 Friedrich-Alexander-Universität Erlangen-Nürnberg, Lehrstuhl für Angewandte Physik, Staudtstr. 5, 91058 Erlangen, Germany; 4 Robert-Koch-Institut, Burgstr. 37, 38855 Wernigerode, Germany; 5 Arbeitsgruppe Mikrobiologie, Universität Osnabrück, Barbarastr. 11, 49076 Osnabrück, Germany

## Abstract

**Background:**

*Corynebacterium diphtheriae*, the causative agent of diphtheria, is well-investigated in respect to toxin production, while little is known about *C. diphtheriae *factors crucial for colonization of the host. In this study, we investigated the function of surface-associated protein DIP1281, previously annotated as hypothetical invasion-associated protein.

**Results:**

Microscopic inspection of DIP1281 mutant strains revealed an increased size of the single cells in combination with an altered less club-like shape and formation of chains of cells rather than the typical V-like division forms or palisades of growing *C. diphtheriae *cells. Cell viability was not impaired. Immuno-fluorescence microscopy, SDS-PAGE and 2-D PAGE of surface proteins revealed clear differences of wild-type and mutant protein patterns, which were verified by atomic force microscopy. DIP1281 mutant cells were not only altered in shape and surface structure but completely lack the ability to adhere to host cells and consequently invade these.

**Conclusions:**

Our data indicate that DIP1281 is predominantly involved in the organization of the outer surface protein layer rather than in the separation of the peptidoglycan cell wall of dividing bacteria. The adhesion- and invasion-negative phenotype of corresponding mutant strains is an effect of rearrangements of the outer surface.

## Background

*Corynebacterium diphtheriae *is the causative agent of diphtheria, a toxaemic localized infection of the respiratory tract. By vaccination diphtheria is well-controlled in e. g. Western Europe [1-3]; however, this disease is still a cause of morbidity and mortality in less developed countries. While the production of diphtheria toxin has been well-established as a major virulence factor, little is known about *C. diphtheriae *factors crucial for colonization of the host and corresponding host receptors recognized by these factors, although colonization is an essential step of pathogenicity.

In the last decades it has become evident that *C. diphtheriae *is not only the aetiological agent of diphtheria, but can cause other infections. Non-toxigenic strains have been increasingly documented [4-6] and found to be the cause of invasive diseases such as endocarditis, bacteraemia, pneumonia, osteomyelitis, spleen abscesses, and septic arthritis ([7] and references therein). These systemic infections caused by *C. diphtheriae *suggest that this pathogen is not only able to attach to host epithelial cells, but must be able to gain access to deeper tissues by unknown portals of entry and to persist in these tissues.

A possible clue for the background of persistence of *C. diphtheriae *came from investigations of adherence and invasion of toxigenic and non-toxigenic strains. Using a combination of gentamicin protection assays and thin-section electron microscopy, Hirata and co-workers [8] showed that toxigenic *C. diphtheriae *were not only able to adhere to laryngeal HEp-2 cells, but also enter these cells and survive after internalization. Similar observations were made for non-toxigenic strains [9] showing that also pharyngeal Detroit 562 cells can be invaded by *C. diphtheriae*. In this study, living intracellular bacteria were detected up to 48 h after infection.

While host cell receptors and invasion-associated proteins of the pathogen are still unknown, bacterial adhesion factors have been recently at least partially characterized on the molecular level. *C. diphtheriae *is able to assemble three distinct pili on its surface. Mutant analyses showed that the SpaA-type pilus is sufficient for adhesion to pharynx cells, shaft proteins are not crucial for pathogen-host interaction, while adherence to pharyngeal cells is greatly diminished when minor pili proteins SpaB and SpaC are lacking [10]. The results obtained in this study also indicated the existence of other proteins besides pili subunits involved in adhesion to larynx, pharynx, and lung epithelial cells, since a total loss of attachment to pharyngeal cells due to mutagenesis of pili- and sortase-encoding genes could not be observed and attachment to lung or larynx cells was less affected by the mutations. This is in line with a number of studies suggesting the multi-factorial mechanism of adhesion (reviewed in [11]). Furthermore, Hirata and co-workers [12] described two distinct patterns of adherence to HEp-2 cells, a localized and a diffuse form, an observation that hint also to the existence of several adhesion factors. This idea is in accordance with the situation in other bacteria such as *Salmonella enterica *where a high number of different factors are crucial for pathogenesis [13]. The involvement of different *C. diphtheriae *proteins to adherence to distinct cell types is further supported by work on adhesion to human erythrocytes, showing that non-fimbrial surface proteins 67p and 72p, which were up to now only characterized by their mass, are involved in this process [14]. Interestingly, besides strain-specific differences in adherences (see references cited above), also growth-dependent effects were observed. In a study using two toxigenic *C. diphtheriae *strains and erythrocytes as well as HEp-2 cells, de Oliveira Moreira and co-workers [15] showed an effect of iron supply on hemagglutination and lectin binding properties of the microorganisms. Also in this study, strain-specific differences in adherence were detected.

While pathogen factors responsible for adhesion are at least partially known, the molecular background of invasion is more or less unclear. Since we were interested in this process, we started a functional genetics approach to identify proteins involved in invasion, based on a recently published work presenting a comprehensive analysis of proteins secreted by *C. diphtheriae *[16]. In this study, we focused on one of these identified proteins, the surface-associated protein DIP1281, a member of the NlpC/P60 family [17]. NlpC/P60 proteins define a large superfamily of several diverse groups of proteins including putative proteases and probably invasion-associated proteins. They are found in bacteria, bacteriophages, RNA viruses, and eukaryotes and various members are highly conserved among non-pathogenic and pathogenic corynebacteria [18]. *C. diphtheriae *protein DIP1281 was, as its homologs Ce1659, Cg1735, and JK0967 in *Corynebacterium efficiens*, *Corynebacterium glutamicum*, and *Corynebacterium jeikeium*, previously annotated as hypothetical invasion-associated protein and was therefore in the focus of this study.

## Results

### Adhesion and invasion of *C. diphtheriae *wild type and mutant strains

As a basis for further analyses of DIP1281 mutants, strains ISS3319 and ISS4060, which were already shown to be adhesion- and invasion-competent [9], were tested for adhesion to and internalization in Detroit562 (D562) cells. Using a slightly modified protocol (compared to [9]) with increased number of washing steps, we were able to generate highly reproducible infection conditions (Table 1). In these experiments, strain ISS3319 showed a higher number of adherent bacteria compared to strain ISS4060 (corresponding to adhesion rates of 2.66 ± 0.12% for ISS3319 and 2.16 ± 0.29% for ISS4060), while statistically relevant differences of the number of invaded epithelial cells were not observed (Table 1).

**Table 1:** Adhesion of *C. diphtheriae *to epithelial cells and internalization. D562 cells (2 × 10^5 ^cells per well) were infected with *C. diphtheriae *(4 × 10^7 ^cfu/ml) leading to a multiplicity of infection (MOI) of 200.

Strain	Viable bacteria (CFU/ml)^a^	
	**adherent^b^**	**internalized^c^**

ISS3319	10.1 × 10^5 ^± 1.4 × 10^5^	1.6 × 10^3 ^± 1.0 × 10^2^

ISS4060	3.5 × 10^5 ^± 1.0 × 10^5^	3.0 × 10^3 ^± 1.4 × 10^3^

Lilo1	1.6 × 10^2 ^± 2.1 × 10^2^	n. d.

Lilo2	9.3 ± 10.6	n. d.

^a ^values represent the means and standard deviations of three separate experiments

^b ^average number of bacteria recovered on agar plates after 1.5 h of infection

^c ^average number of bacteria recovered on agar plates after 1.5 h of infection and further 2 h of treatment with gentamicin

n. d.: not detectable

After establishing infection conditions for the wild-type strains, *dip1281 *gene disruption mutants Lilo1 (ISS3319::pK18 mob*'dip1281''*) and Lilo2 (ISS4060::pK18 mob*'dip1281''*) were analyzed. DIP1281 mutant strains lacked the ability to adhere to host cells almost completely (with adhesion rates of 0.03 ± 0.01% for Lilo1 and 0.04 ± 0.01% for Lilo2) and in contrast to the wild-type no internalized bacteria were detectable for strain Lilo1 and Lilo2 (Table 1).

### Basic characterization of DIP1281 mutant strains

**Figure 1:**
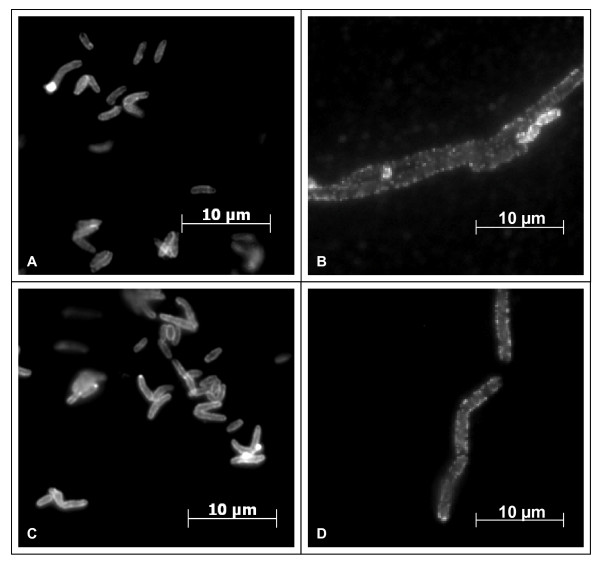
**Immuno-fluorescence microscopy of *C. diphtheriae *wild-type and mutant strains**. An antiserum directed against the surface proteome of *C. diphtheriae *was used as primary antibody; Alexa Fluor 488 goat anti-rabbit was used as secondary antibody. A: ISS3319, B: Lilo1, C: ISS4060, D: Lilo2.

Microscopic inspection of the mutant strains revealed unexpectedly an increased size of the single bacteria in combination with an altered less club-like shape and formation of chains of bacteria rather than the typical V-like division forms or palisades of clustered *C. diphtheriae*. Immuno-fluorescence microscopy carried out for control verified that observation (Figure 1). Additionally, this approach showed an uneven, speckled staining of the mutants, indication an altered surface structure compared to the wild-type strains.

**Figure 2:**
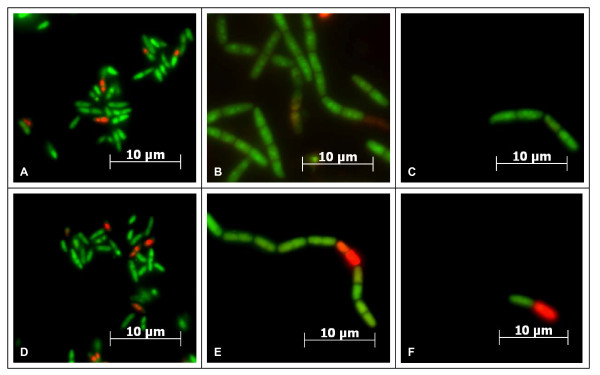
**LIVE/DEAD staining of *C. diphtheriae *wild-type and mutant strains**. Green fluorescent bacteria have a functional cytoplasmic membrane and are stained green, red propidium iodide staining indicates non-viable cells. A: ISS3319, B-C: Lilo1, D: ISS4060, E-F: Lilo2, C and F: cells subjected to 5 min of vigorous vortexing.

To analyse, if all bacteria within the observed chains of mutants were still viable or if changes were correlated with detrimental effects on survival of bacteria, we carried out LIVE/DEAD staining. No significant differences were observed between wild-type and mutants in respect to viability, in all cases the majority of bacteria were fully viable and exclusively stained by SYTO9 green and not by propidium iodide (Figure 2). During manipulation of bacteria (washing steps, resuspension of pellets), we observed that chains of mutants were occasionally broken down to smaller units. Using LIVE/DEAD staining, we could show that disruption of chains by vigorous vortexing (5 min) was not detrimental to the bacteria (Figure 2C and 2F), indicating that mutant strains have a fully functional and rigid peptidoglycan layer.

For all strains, ISS3319, ISS4060, Lilo1 and Lilo2, identical doubling times of about 70 min were observed. Interestingly, with a final optical density (OD_600_) of approx. 13, the mutants reached a more than fourfold higher OD_600 _compared to the corresponding wild-type strains, which reached final optical densities between 2.5 and 3. This observation corresponds nicely with the increased colony size of the mutants (data not shown) and suggests that the altered bacterial size and form has no severe impact on light scattering and consequently OD measurement.

### Analysis of surface proteins

**Figure 3:**
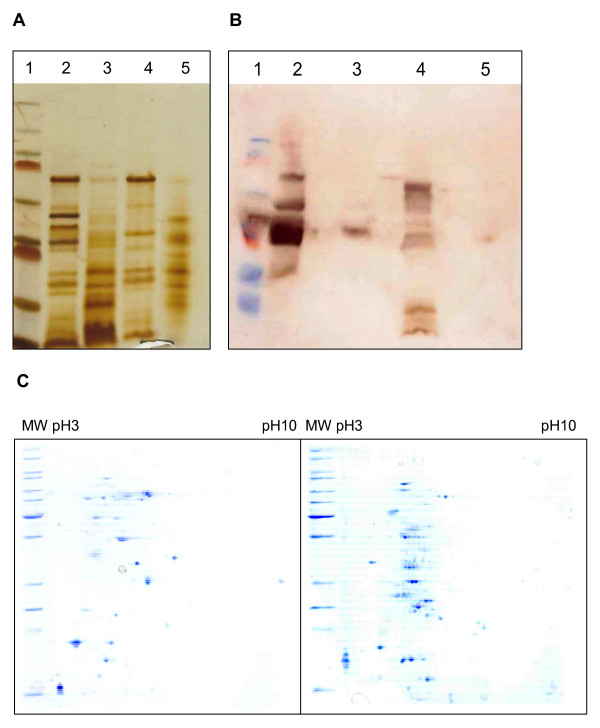
**Analysis of surface proteins**. Surface proteins were isolated from *C. diphtheriae *wild-type and mutant strains and subjected to SDS-PAGE (A), Western blotting (B), and 2-D PAGE (C). For SDS-PAGE 25 μg of protein prepared from strains ISS3319 (lane 2), Lilo1 (lane 3), ISS4060 (lane 4), and Lilo2 (lane 5) were applied per lane on a 10% polyacrylamide gel and silver-stained after electrophoresis. Molecular weight of marker proteins (lane 1, from top to bottom): 250, 130, 95, 72, 55, 36, 28, 17, 11 kDa. Western blotting was carried out after SDS-PAGE using a polyclonal antiserum directed against *C. diphtheriae *DSM44123 surface proteins. For 2-D PAGE surface protein preparations were separated according to their isoelectric point and molecular mass using a pH range of 3-10 for isoelectric focussing and 12.5% polyacrylamide gels for SDS-PAGE. Gels were stained with Coomassie Brilliant Blue. Molecular weight of marker proteins (from top to bottom): 150, 120, 100, 85, 70, 60, 50, 40, 30, 25, 20, 15 kDa.

Since we assumed that the altered shape of the mutants might be correlated with an altered cell surface, especially in the light of the immuno-fluorescence microscopy approach (Figure 1), which showed a different antibody binding compared to the wild-type, we isolated the surface proteins of wild-type and mutant strains. When these were subjected to SDS-PAGE and silver staining, significant differences in protein patterns were observed (Figure 3A). Compared to the protein bands of the wild-type strains, DIP1281 mutants showed a decrease of bands in the upper molecular weight range accompanied by an increased number of bands in the lower molecular weight range. These changes were confirmed, when Western blot experiments were carried out (Figure 3B), which also showed a dramatic change and decrease of immuno-reactive bands. As a third experimental approach to analyse surface proteins, 2-D PAGE was carried out (gels for strains ISS3319 and Lilo1 are shown in Figure 3C; ISS4060 and Lilo2 gave comparable results, data not shown). As in the SDS-PAGE experiments, the mutant showed a decrease of proteins in the upper molecular weight range and an increased number of spots in the lower molecular weight range. Furthermore, in comparison to the wild-type, the mutant showed a dramatic increased number of multiple spots. The molecular background of these multiple protein forms is unclear.

### Surface structure of wild-type and mutant strains

**Figure 4:**
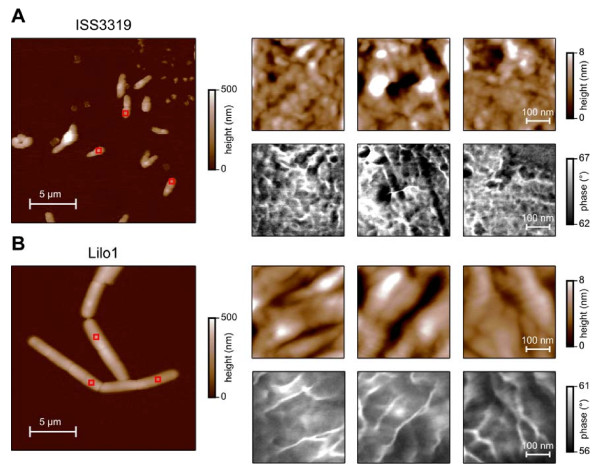
**Ultrastructural analysis of the cell surface of *C. diphtheriae *strains**. (A) ISS3319, (B) Lilo1; red boxes in the low magnification images on the left hand side mark three areas shown with a higher magnification on the right hand side (upper row: topography/height, lower row: phase). Colour scale bars at the right hand side give height and phase magnitudes.

The altered immuno-staining of the mutant strain surfaces and the clear differences of wild-type and mutant protein patterns revealed by SDS-PAGE and 2-D PAGE prompted us to perform a more detailed investigation of the cell surface of *C. diphtheriae *by atomic force microscopy. Compared to the surface structure of *C. glutamicum*, which was investigated for several strains in great detail by atomic force microscopy [19-21], *C. diphtheriae *shows a more structured surface (Figure 4). Furthermore, striking differences were observed when the cell surface of different *C. diphtheriae *strains was examined. In the wild-type strain ISS3319 (Figure 4A) round elevations with a lateral diameter of 10-40 nm and a height of 1-4 nm can be seen (Figure 4A, upper row). The complementary phase images, which reflect adhesive and elastic tip-sample interactions, show a similar, highly structured surface structure (Figure 4A, lower row). In the mutant strain Lilo1 (Figure 4B), a loss of this fine structure was observed: Elongated elevations can be seen with a width of 50-100 nm (Figure 4B, upper row). Their height is similar as in the case of the wild-type strain. Differences in surface structure are especially obvious when comparing the complementary phase images (lower rows in Figure 4A and 4B). Analyses of strains ISS4060 and Lilo2 gave similar results (data not shown).

## Discussion

In this study, the function of the surface-associated protein DIP1281, a member of the NlpC/P60 family was investigated, which was annotated as hypothetical invasion-associated protein. By fluorescence staining and atomic force microscopy, we could show that DIP1281 mutations cause formation of chains of bacteria, rearrangements of cell surface structures, and dramatic changes in protein patterns. Our data indicate that DIP1281 is not crucial for the separation of the peptidoglycan layer of dividing bacteria, since disruption of chains did not decrease the viability of bacteria. Consequently, DIP1281 function seems to be limited to the outer protein layer of *C. diphtheriae*, which is not uniformly organized in a surface layer lattice, but comprises more than 50 different proteins [16]. If the other NlpC/P60 family members in *C. diphtheriae *besides DIP1281, namely DIP0640, DIP1621, and DIP1622 [18] have similar functions in cell surface layer organization is unknown and has to be investigated in future projects.

Tsuge and co-workers reported cell separation defects in *Corynebacterium glutamicum *R, when the DIP1281 homolog cgR_1596 and another member of the NlpC/P60 protein family cgR_2070 were mutated [22]. Also in this study, cell separation was not impaired in respect to separation of peptidoglycan and mycolic acid layers of daughter cells, but mainly restricted to the surface protein layer of the bacteria. However, using transmission electron microscopy of thin sections of cells, in this study also formation of multiple septa within single bacteria was observed in response to *cgR_1596 *mutations. Furthermore, growth of mutant strains was examined. In contrast to the situation in *C. diphtheriae*, where we found an unaltered growth rate and a strongly increased biomass formation caused by lack of DIP1281, in *C. glutamicum R *mutation of *cgR_1596 *led to a slightly decreased growth rate and unaltered final optical density of the culture. The exact function of the NlpC/P60 protein family members in *C. glutamicum *was also not unravelled until now.

In respect to adhesion and internalization of *C. diphtheriae *to epithelial cells, the results obtained in this study suggest that DIP1281 is crucial for localization and function of adhesion and invasion factors and consequently, structural alterations caused by lack DIP1281 prevent adhesion of corresponding mutants to host cells and invasion into these cells.

## Conclusions

*C. diphtheriae *protein DIP1281 was, as its homologs Ce1659, Cg1735, and JK0967 in *Corynebacterium efficiens*, *Corynebacterium glutamicum*, and *Corynebacterium jeikeium*, previously annotated as hypothetical invasion-associated protein. Generation and analyses of mutant strains indicate that DIP1281 is predominantly involved in the organization of the outer surface protein layer of *C. diphtheriae *rather than in the separation of the peptidoglycan cell wall of dividing bacteria. The adhesion- and invasion-negative phenotype of corresponding mutant strains is an effect of rearrangements of the outer surface of bacteria. Specific interaction partners for DIP1281 and its homologs in other corynebacteria are unknown and might be the focus of further studies to unravel the specific functions and targets of these proteins on a molecular level.

## Methods

### Bacterial strains and growth

Strains used in this study are listed in Table 2. *Escherichia coli *DH5αMCR was grown in Luria Bertani (LB) medium at 37°C, *C. diphtheriae *in Heart Infusion (HI) broth at 37°C. If appropriate, kanamycin was added (30 μg/ml for *E. coli*; 50 μg/ml for *C. diphtheriae*).

**Table 2:** Bacterial strains and eukaryotic cells used in this study.

Strains	Description	Reference
***C. diphtheriae***		

DSM44123	non-toxigenic isolate, type strain	DSMZ (Braunschweig)

ISS3319	*C. diphtheriae *var. *mitis*, non-toxigenic isolate	[9]

ISS4060	*C. diphtheriae *var. *gravis*, non-toxigenic isolate	[9]

Lilo1	ISS3319 DIP1281::pK18*mob*'*DIP1281*''	This study

Lilo2	ISS4060 DIP1281::pK18*mob*'*DIP1281*''	This study

***E. coli***		

DH5αMCR	*endA1 supE44 thi-1 *λ^- ^*recA1 gyrA96 relA1 deoR Δ(lacZYA-argF) U196 φ80ΔlacZ ΔM15 mcrA Δ(mmr hsdRMSmcrBC)*	[28]

**Cell lines**		

Detroit562	human hypopharyngeal carcinoma cells	[29]

### Preparation of *C. diphtheriae *protein extracts

To prepare surface proteins, bacteria were grown in 20 ml HI broth (with kanamycin added for the mutant strains) for approximately six hours and used to inoculate 250 ml HI broth for overnight growth. Bacteria were harvested by centrifugation at 5,000 × g for 20 min, washed twice with pre-cooled (4°C) 50 mM Tris-HCl buffer (pH 7.2), resuspended in 50 mM Tris-HCl (pH 7.2) containing 2% 3-[(3-choamidopropyl)-dimethylammonio] propanesulfonate (CHAPS) and incubated on ice overnight, followed by centrifugation at 3,500 × g and 4°C for 30 min to separate the cell surface proteins. After filtration of the protein solution using 0.45 μm pore-size filters (SARSTEDT, Nümbrecht, Germany), further preparation of the surface proteins by phenolic acid extraction and methanol precipitation followed a protocol described by Watt and co-workers [23]. The precipitated proteins were harvested by centrifugation at 3,500 × g and 4°C for 30 min. The pellet was washed twice with 3 ml of 70% ethanol (-20°C) and once with 3 ml of acetone (-20°C). Finally, the protein pellet was dried on ice and solubilised in 450 μl of dehydration buffer (8 M urea, 20 mM DTT, 2% CHAPS). Protein concentrations were determined spectrophotometrically using a NanoDrop spectrophotometer (peqLab, Erlangen, Germany). Surface proteins prepared from strain DSM44123 were used for the immunization of rabbits to generate *C. diphtheriae *surface protein-specific antisera (Eurogentec, Liege, Belgium).

### SDS-PAGE, silver staining, and Western blot analysis

Proteins of the cell surface fraction of wild-type and mutant strains were separated using Tricine-buffered 10% SDS gels as described [24]. After SDS-PAGE protein bands were visualized by silver staining [25]. For Western blotting, the SDS gel-separated proteins were transferred onto a polyvinylidene difluoride membrane by electroblotting (PVDF, Roth, Karlsruhe, Germany) and incubated with *C. diphtheriae *surface protein-specific antisera generated in rabbits. Antibody binding was visualized by using goat anti-rabbit IgG coupled to alkaline phosphatase and the BCIP/NBT alkaline phosphatase substrate (Sigma-Aldrich, Darmstadt, Germany).

### 2-D-PAGE of *C. diphtheriae *surface proteins

2-D polyacryalmide gels were loaded with 300 μg of proteins dissolved in 450 μl of solution B (8 M urea, 20 mM DTT, 2% CHAPS, a trace of bromophenol blue, and 0.5% Pharmalyte 3-10). IEF was performed with commercially available IPG strips (18 cm, pH 3-10) and the Ettan IPGphor II (GE Healthcare, Munich, Germany). The following voltage profile was used for IEF: 1 h, 0 V; 12 h, 30 V; 2 h, 60 V; 1 h, 500 V; 1 h, 1000 V followed by a linear increase to 8000 V. The final phase of 8000 V was terminated after 90,000 Vh. The IPG strips were equilibrated for 30 min each in 5 ml of solution C (6 M urea, 50 mM Tris-HCl (pH 6.8), 30% glycerol, 2% SDS, 1% DTT) and in 5 ml of solution D (6 M urea, 50 mM Tris-HCl (pH 6.8), 30% glycerol, 2% SDS, 4% iodacetamide). The isolated proteins were separated in 12.5% acrylamide/bis-acrylamide gels (37.5:1) with an Ettan Dalt II system (GE Healthcare, Munich, Germany) applying approximately 15 mA per gel. To visualize the separated proteins, gels were stained in Coomassie staining solution (5% methanol, 42.5% ethanol, 10% acetic acid, 0.25% Serva-G250), and destained with 10% acetic acid.

### Immuno-fluorescence

For immuno-fluorescence staining a rabbit antiserum directed against the *C. diphtheriae *surface proteome was used as primary antibody. As secondary antibody Alexa-Fluor 488 (green) goat anti-rabbit IgGs were applied. All antibodies were diluted in blocking solution (2% goat serum, 2% BSA). Bacterial cells were dried on coverslips (37°C), fixed with 3% PFA (10 min at room temperature) and finally washed thrice with 1 × PBS. Bacterial cells were incubated in staining solution for at least 1 h at room temperature and washed thrice with PBS between staining steps. Coverslips were mounted on glass slides using Fluoroprep (Biomerieux, Craponne, France). Imaging was done on an AxioVert 200 M inverted optical microscope (Carl Zeiss Micromaging GmbH, Jena, Germany). For additional image processing Photoshop CS2 (Adobe, Munich, Germany) was used.

### LIVE/DEAD staining

Overnight cultures grown in 20 ml HI broth plus kanamycin added for the mutant strains were washed once in 20 ml 1 × PBS and resuspended in 10 ml 1 × PBS. To distinguish live and dead bacteria the *LIVE/DEAD Bac*light Bacterial Viability Kit for microscopy (Invitrogen, Eugene, OR) was used according to the supplier's protocol. Imaging was done on an AxioVert 200 M inverse microscope (Carl Zeiss Micromaging GmbH, Jena, Germany).

### Atomic force microscopy (AFM)

Overnight cultures grown in 20 ml HI broth plus kanamycin added for the mutant strains were washed five times in 20 ml ice cold distilled water and finally resuspended in 10 ml ice cold distilled water. 5 μl of each sample were fixed on a glass slide by drying using compressed air. An AFM instrument (MFP-3D, Asylum Research, Santa Barbara, CA) with standard silicon cantilever probes (NCH-W, Nanosensors, Neuchatel, Switzerland) was used under ambient laboratory conditions and operated in tapping mode. AFM topography and phase images were recorded simultaneously.

### Adhesion assays

D562 cells were seeded in 24 well plates (Greiner bio-one Cellstar, Frickenhausen, Germany) at a density of 2 × 10^5 ^cells per well 48 h prior to infection. Bacteria were inoculated to an OD_600 _of 0.1 from overnight cultures and grown in HI broth for 3.5 h. Subsequently, the bacteria were harvested by centrifugation and adjusted to an OD_600 _of 0.2. A master mix of the inoculum was prepared in DMEM (Dulbecco's modified Eagle's medium, PAA; high glucose, 10% FCS, 2 mM glutamine) without penicillin/streptomycin and cells were infected for 90 min at a MOI of 200 (viable counts experiments). The cells were washed with PBS nine times, detached with 500 μl trypsin solution (0.12% trypsin, 0.01% EDTA in PBS) per well (5 min, 37°C, 5% CO_2_, 90% humidity) and lysed with 0.025% Tween 20 for 5 min at 37°C. Serial dilutions were made in pre-chilled 1 × PBS and plated on HI plates to determine the number of cfu. The assay is modification of a previously described one [9].

### Epithelial cell invasion model

D562 cells were seeded in 24 well plates (Greiner bio-one Cellstar, Frickenhausen, Germany) at a density of 2 × 10^5 ^cells per well 48 h prior to infection. Overnight cultures grown in HI were re-inoculated to an OD_600 _of 0.1 in fresh medium and grown aerobically for another 3.5 h. An inoculum of approximately 8 × 10^7 ^bacteria ml^-1 ^(MOI = 200) was prepared in DMEM without penicillin/streptomycin and 500 μl per well were used to infect the D562 cells. The plates were centrifuged for 5 min at 500 × g to synchronize infection and subsequently incubated for 90 min (37°C, 5% CO_2_, 90% humidity). The cells were washed thrice with PBS and 500 μl of DMEM containing 100 μg ml^-1 ^gentamicin was applied to each well to kill remaining extracellular bacteria. After 2 h of incubation the cell layers were washed thrice with PBS, detached by adding 500 μl trypsin solution (0.12% trypsin, 0.01% EDTA in PBS) per well (5 min, 37°C, 5% CO_2_, 90% humidity), and lysed for 5 min at 37°C with 0.025% Tween 20 to liberate the intracellular bacteria. Serial dilutions of the inoculum and the lysates were plated on HI plates to determine the number of colony forming units (cfu).

### Construction of mutant strains

For plasmid isolation, transformation and cloning, standard techniques were used [26]. For chromosomal disruption of the *C. diphtheriae DIP1281 *gene an 582 bp internal DNA fragment was amplified *via *PCR using chromosomal DNA of strain ISS3319 as template and the following primers: 5'- cgc gcg **ctc gcg **ggc acg tca gga agc tg - 3'; 5'- cgc gcg **ccc ggg **cga atc caa ttt tat taa aa - 3'. Using the *Ava*I and *Xma*I sites introduced in *via *the PCR primers (shown in bold) the DNA fragment was ligated to *Ava*I/*Xma*I-restricted and dephosphorylated pK18 mob DNA [27]. The resulting plasmid pK18 mob*DIP1281*' was amplified in *E. coli *DH5αMCR. One microgram of unmethylated plasmid isolated from this *E. coli *strain was used to transform *C. diphtheriae *using a GenePulser II (Bio-Rad, Munich Germany). Electroporated cells were added to 1 ml of HI broth containing 1% glucose and incubated for 2 h at 37°C. An appropriate volume of culture was plated on medium containing kanamycin. Since pK18 mob cannot be replicated in *C. diphtheriae*, kanamycin-resistant *C. diphtheriae *carried the vector integrated *via *recombination in the chromosomal *DIP1281 *gene and were designated Lilo1 (resulting from the strain ISS3319) and Lilo2 (resulting from the strain ISS4060).

## Authors' contributions

LO carried out growth mutagenesis experiments, invasion assays, fluorescence microscopy, protein preparation and analysis, MHö carried out adhesion experiments, RGG and MHe supported LO and MHö in respect to cell culture, adhesion and invasion analysis and fluorescence microscopy, AFM experiments were carried out in cooperation with JR and TES, AB supervised the experiments of LO and MHö and was responsible for the draft and final version of the manuscript. All authors read and approved the final manuscript.
